# Independent risk factors for long-term mortality in patients with severe infection

**DOI:** 10.1186/cc14096

**Published:** 2015-03-16

**Authors:** J Francisco, I Aragão, T Cardoso

**Affiliations:** 1 Centro Hospitalar do Porto - Hospital Geral de Santo António, Porto, Portugal

## Introduction

The purpose of this study was to examine long-term mortality, 5 years after severe infection, and to identify independent risk factors associated with it.

## Methods

A prospective cohort study developed at a tertiary care university-affiliated 600-bed hospital including all patients with severe infection admitted into intensive care, medical, surgical, haematology and nephrology wards, over a 1-year period (2008/2009).

The outcome of interest was mortality 5 years following hospitalisation and its association with specific risk factors was studied through logistic regression.

## Results

**Figure 1:**
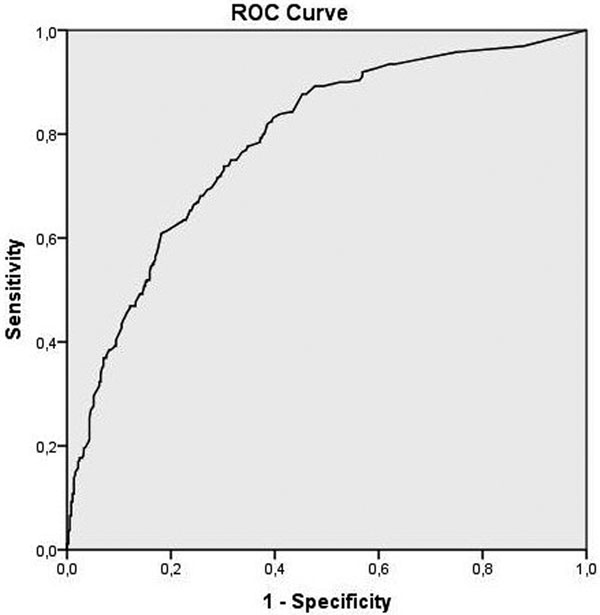


There were 1,013 patients included in the study. Hospital mortality rate was 14% (*n *= 137) and 5-year mortality was 37% (*n *= 379). Factors independently associated with 5-year mortality were (adjusted odds ratio (95% confidence interval)): age = 1.04 per year (1.03 to 1.05), cancer = 8.00 (3.06 to 20.88), chronic hepatic disease = 3.06 (1.06 to 8.87), chronic respiratory disease = 2.21 (1.06 to 4.62), haematologic disease = 3.40 (1.64 to 7.04), Karnovsky Index <70 = 2.56 (1.63 to 3.71), infection by an ESKAPE pathogen = 1.65 (1.02 to 2.66) and severity of infection (reference is infection without SIRS): sepsis = 1.14 (0.7 to 1.83), severe sepsis = 1.18 (0.73 to 1.93), septic shock = 3.69 (1.78 to 7.65). The final model had a very good discrimination for long-term mortality with an area under the ROC curve of 0.78 (Figure 1).

## Conclusion

The authors identified several factors that were significantly associated with increased long-term mortality in patients with severe infection. This information will help clinicians in the discussion of individual prognosis and clinical decision-making.

